# Is routine prenatal screening and testing fundamentally incompatible with a commitment to reproductive choice? Learning from the historical context

**DOI:** 10.1007/s11019-020-09985-w

**Published:** 2020-10-30

**Authors:** Panagiota Nakou

**Affiliations:** Department of Law, School of Social Sciences, Faculty of Humanities, The University of Manchester, Williamson Building, Oxford Rd, Manchester, M13 9PL UK

**Keywords:** Prenatal screening, Prenatal diagnosis, Routinisation, Reproductive choice, Reproductive autonomy, Bioethics

## Abstract

An enduring ethical dispute accompanies prenatal screening and testing (PST) technologies. This ethical debate focuses on notions of reproductive choice. On one side of the dispute are those who have supported PST as a way to *empower* women’s reproductive choice, while on the other side are those who argue that PST, particularly when made a routine part of prenatal care, *limits* deliberate choice. Empirical research does not resolve this ethical debate with evidence both of women for whom PST enhances their choices but also persistent evidence of recurrent problems between PST and women’s autonomous decision-making. While there have been attempts to remove challenges to reproductive choice, it has been argued that these challenges cannot be removed entirely. In this paper I provide a historical review of PST technologies’ development and in doing so provide a detailed insight into the root causes of this tension between the opposing sides of this debate. This historical account provides evidence that those who championed the early use of these technologies did so in order to achieve a number of wholly different goals other than women’s choice and empowerment. These different aims focus on scientific discovery and eugenic goals and, I argue, are *irreconcilable* with women’s choice and empowerment. It thus may not be surprising that the resulting practice of PST continues to resist compatibility with women’s choice and empowerment. Ultimately, by understanding the historical foundations of PST we can more effectively assess how to *reconcile* women’s reproductive autonomy with routine prenatal screening.

## Introduction

Routine prenatal screening is an integral part of most modern healthcare systems. This kind of routine prenatal screening is typically the process where women are routinely offered screening tests for conditions such as aneuploidies as part of their antenatal care with an implicit recommendation that they accept these offers in most cases (Bennett 2001, pp. 463–464; Suter 2002, pp. 241–242; NICE 2008; Kater-Kuipers et al. 2018, p. 626). This is not mandatory screening but involves making these screening tests a routine part of antenatal care for all women accessing healthcare. Looking at healthcare systems in the western world and particularly, in Europe and the US which constitute the focus of this work, one can observe that despite the “national variations” (Vassy et al. 2014, p. 73) of screening strategies, prenatal screening and testing (PST)^1^ technologies have been an indispensable part of prenatal care likely to be offered in a routine manner.

In particular, the clinical use of several types of PST technologies differs from time to time and among different jurisdictions and countries. For instance, PST can be part of a prenatal care system funded by the state or it might be accessible through different systems which are in place in private healthcare settings; The types of PST technologies and the way these become available to the public can differ not only among different countries but also from place to place in the same country; The reasons and criteria behind the choice of particular prenatal screening strategies may differ too (Boyd et al. 2010). Nevertheless, apart from these variations, the widespread use of PST technologies and their routine offer has been a common place for many years and in many countries with different healthcare systems.

Looking at the more recent past for instance, between 2001 and 2010 we encounter similar clinical practice guidelines in France, the UK, Australia, New Zealand, Canada and the US, recommending “that all pregnant women be offered prenatal screening” (Vassy et al. 2014, p. 69; Pioro et al. 2008, pp. 1027–1028; Tapon 2010, p. 114). Looking at Europe, in a study of 2004 which aimed “to ‘map’ the […] state of prenatal screening and diagnosis in 18 countries in Europe that are members of EUROCAT” (Boyd et al. 2008, p. 690), it was found that “the majority of countries had moved from solely offering older mothers a diagnostic test to having some form of Down’s syndrome screening in place, with over half having an official country-wide policy or recommendation for first or second-trimester screening” (Boyd et al. 2008, p. 693). Similar observations can be made by reading the equivalent special report of EUROCAT in 2010 (Boyd et al. 2010). What is more interesting is that the adoption of the wide use and routinised offer of some kind of PST technology is not encountered only in one type of system, such as a publicly funded system. For instance, in the UK prenatal screening and testing is part of a publicly funded healthcare system, France has adopted a reimbursement scheme (Vassy et al. 2014, p. 69) and in the US, although there is no unified guidance and PST technologies constitute private options “more determined by individual preference and health insurance” (Tapon 2010, p. 120), the idea of routine is well established (Hunt 2000; Staff reporter-genomeweb 2020).

But how did we get to this point where this screening has become an often unquestioned and integral part of healthcare and why might looking back at the origins of PST be important to the current ethical debate around routine PST?

The development, establishment and continued provision of PST is regularly characterised as being motivated by women’s demands. One typical example can be seen in a letter by two gynaecologists in the late 1980s where they emphasise that “[p]eople want to start now” (Harbers 2005, p. 241). The claim that the introduction of prenatal screening was motivated by women’s demands (Powledge 1979, p. 16; Löwy 2014, p. 293), and further justified in terms of choice empowerment, has been used by clinicians, the media and the scientific literature to support the introduction and expansion of PST since the 1970s (Shakespeare 2011, p. 38). However, while those promoting PST have claimed that these techniques empower women over reproductive choice, there has been a great deal of criticism of this claim too (Suter 2002; Bennett 2001; Lippman 1991; Tymstra 1991). Those who criticise it, argue that the reality of PST is that its offer is one that is very difficult to refuse (Schmitz et al. 2009; Wahlberg and Gammeltoft 2018, p. 78), and where prenatal screening is part of routine prenatal care, deliberate choice is limited (Suter 2002, pp. 254–255).

Recent developments, such as the introduction of Non-Invasive Prenatal Testing (NIPT) into existing routine screening, have fanned the flames of a longstanding debate concerning the challenges that routine prenatal screening presents to women’s autonomous choice (Kater-Kuipers et al. 2018; Deans et al. 2013; van den Heuvel et al. 2010). The example of NIPT shows that, notwithstanding continuing attempts to improve the quality of women’s choices when it comes to PST (Nuffield Council on Bioethics 1993, 2017, 2018, Gregg et al. 2013, p. 396; Benn et al. 2013, p. 623; Harcombe and Armstrong 2008, pp. 579–581), the concerns persist (Hyacinth 2017; Brownsword and Wale 2018; Vanstone et al. 2018; Cernat et al. 2019). It has been argued that the problem of the quality of consent given during PST is one that ultimately may not be able to be solved due to a fundamental incompatibility between women’s choices and routine PST (Bennett 2007). It is this claim that this paper aims to explore. However, instead of exploring it in the context of current PST, this paper takes a different approach. This different approach pertains to an exploration of the history of PST in order to gain an understanding of the root causes of why the concerns about the choice to engage in screening for disability persist despite well-meaning attempts to mitigate these concerns.

As such, this paper provides a detailed overview of the historical events that influenced the emergence, development and establishment of PST. At this point I should clarify that given that the wide diffusion of PST use and the involvement of some kind of routine offer concern a variety of healthcare systems, in this work I do not examine the case of the routinisation of PST in a particular healthcare system. Also, this work is not a critique of the various reasons and interests (e.g. resource allocation, cost effectiveness, disability care, access to abortion, other aims) that may justify or deny the establishment of a routine PST system in a healthcare system (Vassy et al. 2014, p. 68; Gilbert et al. 2001; Boyd et al. 2010); I do not examine the routinisation of PST as a ‘problem’ that needs to be solved. Through a historical review I attempted to find out the extent to which the wide and routine use of PST was based on the aim to empower women’s reproductive choice. Therefore, this historical account provides evidence that women’s choice was not a primary motivator of PST development and expansion. In fact, there is strong evidence to infer that those who championed the early use of these technologies were motivated by further scientific discovery, eugenic goals and financial profit rather than women’s choice and empowerment. Although this evidence does not offer a solution to this longstanding ethical debate, it is important that it is acknowledged as by understanding what motivated the development of PST we can understand why it has been difficult to improve the quality of women’s consent in this context. By this, I do not mean to say that routine PST should necessarily be abandoned. Instead, I am arguing that, given the historical foundations of PST it will continue to be extremely difficult to base the justification of it clearly on women’s choice and empowerment. Ultimately, I suggest that if we really do value women’s choice and empowerment, then understanding the origins of PST will enable us to develop policies that really do put women’s choices at the centre of these endeavours.

## The role of a historical review in the debate around the ethics of women’s reproductive choices within routine PST

A number of different time periods in the nineteenth century have been touted as the ‘birth’ of modern prenatal screening. For instance, it has been argued that the introduction of amniocentesis in the 1950s signaled the beginning of the use of prenatal screening technologies (Ettorre 2001, p. 38). Similarly, the 1960s has been characterised as the decade which gave birth to the contemporary prenatal diagnosis (Löwy 2017, p. 43). The 1970s has also been identified as crucial in the development of PST as the point where medical innovations such as amniocentesis, the study of human chromosomes and obstetrical ultrasound, were first available alongside the legalisation of abortion (Löwy 2014, p. 290). Whether we hold that modern PST was ‘born’ in the 1950s, 1960s or 1970s, these different claims about the origin of PST all have some common ground. What these claims have to unite them is that they argue that, since the end of the 1950s, the clinical use of PST began and expanded as a response to women’s demand and it was morally justified as a way to empower women and their reproductive choices (Seavilleklein 2009, p. 73; Vassy 2006, p. 2043; Cowan 1993, pp. 10–17).

Respect for autonomy and the empowering of individuals to make choices about their lives has been a central principle of modern medical ethics since around the 1970s (Saad 2018, p. 125; Morley and Floridi 2019, p. 1160) and as a result, notions of medical coercion have become not just unpopular, but in most instances, unjustifiable. As a result, it is easy to see why basing the justification of PST on respecting and enhancing autonomy might have a strong appeal. According to the autonomy oriented approach “access to prenatal testing supports and promotes women’s informed choices, empowering them to manage their pregnancies—and hence their lives—in ways that align with their preferences and values” (Ravitsky 2017, p. S34). Similarly, Stapleton argues that “prenatal screening is […] aimed at empowering couples with sufficient capabilities for making meaningful reproductive choices” (Stapleton 2017, pp. 203–204). Moreover, it has been acknowledged that the major aim of prenatal screening is the promotion of informed choices (Williams et al. 2002, p. 743; John 2015) or, in other words, the major aim of offering prenatal screening is to enable “meaningful reproductive choices” (De Jong et al. 2011, p. 657)*.* This approach is reflected in the language used to justify the use PST, where it is invariably suggested that PST promotes, enables, empowers or, similarly, strengthens, facilitates, increases or enhances women’s reproductive choices. In essence, the claim is that the substantial aim of using these technologies is to uphold the idea of reproductive autonomy by empowering women-in terms of enabling and liberating them-to make their own authentic choices about reproduction.

Considering that this aim has morally justified the introduction and wide use of PST technologies in theory, here I examine the extent to which this justification has been in fact the foundation of the introduction and the wide use of PST. In other words, by looking at the historical past of these technologies, I intend to find out whether their introduction and wide use was a response to women’s demands and truly served the substantial meaning of the idea of empowering women’s reproductive choice.

In particular, I examine the period of the emergence of prenatal diagnosis techniques, the influential role of eugenics in their development, the historical events that influenced the trajectory of PST after the 1960s, and the role that governmental, scientific and clinical intentions have played towards the expansion of PST use. The reason why I look to history to put forward a moral argument is reflected in the following quote by Montgomery:

Bioethics governance should provide a process for truth and reconciliation in relation to past failures […] [a] work which requires detailed documentary analysis, judgments on personal responsibility and liability, and historical insight to avoid anachronistic assessments (2017, pp. S25–S26).

Following Montgomery’s approach, in the subsequent sections, I consider the many attempts to reconcile women’s autonomy and empowerment with prenatal screening. I argue that by identifying the actual rationales and goals that motivated PST, that often have very little to do with women’s choice, we can begin to better understand why it has been so difficult to reconcile women’s choice with PST. By understanding past failures to safeguard reproductive choice within routine PST plans, I attempt to identify the root causes of such failures. In particular, I show that given the lack of consideration of women’s choices in the foundations, the development, the introduction and the subsequent routinisation of PST, it is not surprising that the resulting practice continues to resist compatibility with women’s choice and empowerment. This observation is a lesson to be learnt from the past which helps in the understanding of perpetual challenges when it comes to women’s autonomous choices about prenatal screening and testing. Hence, the historical overview that follows could be considered as a contribution to “the process for truth and reconciliation in relation to past failures” (Montgomery 2017, pp. S25–S26) to safeguard and serve the essential meaning of women’s choice empowerment in the case of PST. By understanding the historical foundations of PST we can more effectively assess how to reconcile women’s reproductive autonomy with routine prenatal screening.

## The dawning of prenatal diagnosis techniques until the 1960s: science and eugenics were in action while women’s choice was on mute

In the previous section, I noted that there is a common claim encountered in the literature suggesting that PST was developed and introduced in response to women’s demand. Although below I challenge this claim, before we move onto this challenge let us assume for now that this claim is true. This leaves us with a reasonable question: if women’s requests for access to prenatal diagnostic technologies initiated these technologies’ introduction and expansion, did women’s requests initiate the scientific processes for PST technological invention too? The historical analysis that follows provides evidence that this technological invention was the result of many years of scientific research; whereas this research was often supported and promoted by eugenics, a women’s demand and the idea of women’s choice were absent.

### Scientific trajectory

Looking at the historical literature, we learn that since before the 1940s there had been research and scientific interest in prenatal diagnosis (Suter 2002, p. 234; Löwy 2017, p. 43; Casper 1998, pp. 30–72). For example in the 1930s, we encounter the famous study by Dr. Penrose, who had observed the significant relation between increasing maternal age and birth of Down syndrome (DS) offspring (Penrose 1938; Russo and Blakemore 2014) which set the basis for the development of ‘the primary method to identify women at risk for aneuploidy’ (Russo and Blakemore 2014, p. 183).

In particular, there is evidence about the technology of prenatal diagnosis suggesting that this did not occur as a response to women’s demand. It mostly constituted the development of clinical practice, namely the amniotic tap, which, since the end of the nineteenth century, had been used for entirely different obstetric purposes than prenatal diagnosis (Cowan 2008, pp. 74–75). Neither did this technology develop as a response to a women’s demand for prenatal diagnosis of aneuploidies, such as DS, although later, DS became the focus of massive PST strategies allegedly aiming to satisfy women’s demand. In fact, the successful efforts since the 1940s to understand the causes of Rh disease (a condition where the expectant mother’s antibodies damage the fetuses red blood cells causing developmental impairments) resulted in Bevis’ confirmation that “in pregnancies at risk for Rh disease, the extent of damage to the fetus could be estimated by optical examination of amniotic fluid obtained by amniotic tap before birth” (Cowan 2008, p. 76). Eventually, in the late 1950s, after the observation that the amniotic tap could be used as a diagnostic tool, the technique was renamed to amniocentesis, the application of which would aim for prenatal diagnosis, an entirely different goal than the technique’s primary goal (Cowan 2008, pp. 76–78).

Effectively, there is evidence that the obstetric world was experimenting with prenatal diagnosis alongside the needs of clinical practice. However, there is not clear evidence of these developments being motivated by women’s demands. It seems that prenatal diagnostic technology was a fortunate finding rather than a response to a demand. Yet, while there is no indication of women’s demand during this period, the invention of PST technologies and their subsequent extensive use cannot be attributed to scientific interest and curiosity or coincidence only. In this period there is significant evidence about the influence of eugenics, although the movement’s decline had begun.

### Eugenics until the 1960s

The rise and peak of the modern eugenics movement, “a form of social engineering than a science” (Suter 2002, p. 234), came in the late nineteenth and early twentieth century (Kerr and Shakespeare 2002, pp. 13–19). In Britain, Mazumdar informs us that “[t]he eugenic problematic had grown out of the union of a middle-class activism focused upon the pauper class, with a biological view of human failings” (1992, p. 258). The decline of eugenics’ popularity began in the 1930s and intensified after the Second World War’s Nazi atrocities. This change has also been attributed to this period’s economic and political changes (Kerr and Shakespeare 2002, p. 62); “In the egalitarian world of welfare and economic growth, the pauper class had disappeared. A class analysis no longer carried weight, and with the loss of the class dimension the eugenic problematic could no longer survive in its original form” (Mazumdar 1992, p. 258). A third reason for the movement’s decline was the realisation that eugenics’ rationale about human deficiencies was scientifically wrong (Kerr and Shakespeare 2002, p. 64). During the eugenics decline period, scientific research, such as the famous study by Penrose, on maternal age and increased chance of Down’s syndrome (Penrose 1938), undermined the more extreme aspects of eugenics narrative and many scientists including Penrose had been criticising the movement’s concept (Kerr and Shakespeare 2002, p. 64).

Nevertheless, Penrose’s famous study was partly funded by the Eugenics Society and Penrose himself, although he disagreed with eugenics, was motivated by the “eugenic problematic” (Mazumdar 1992, p. 258 in Kerr and Shakespeare 2002, p. 63). Generally, in the 1930s and 1940s, the majority of human genetics sponsors were motivated by eugenics. Apparently, despite the decline, eugenics remained active in the 1940s when we also encounter a new move towards reform eugenics “aiming to achieve the best children possible” (Kerr and Shakespeare 2002, p. 65). This move remained in the 1950s when links between human genetics and eugenics were still encountered. For example, Kerr and Shakespeare note that during the 1950s “five out of six presidents of the American Society of Human Genetics were also members of the Eugenics Society’s board of directors,” as well as, that “genetic clinics were often […] eugenically inspired” and “various important geneticists also applauded the eugenic significance of genetic clinics” (2002, p. 67).

Thus, evidently, amniocentesis, which was first developed in the 1950s (Suter 2002, p. 235) and recognised as a prenatal diagnostic technique by the end of the same decade (Cowan 2008, p. 78), emerged in the same period that eugenics rhetoric and influence were still active. Consequently, it would be an oversight to claim that prenatal diagnosis that came to the forefront this period was left uninfluenced by eugenics ideology, even if influence was exerted in a more muted and implicit way (Kerr and Shakespeare 2002, p. 77).

A particular example proving that such influence existed is the one of a Danish Act of Parliament which in 1956 legalised “eugenic” abortion after prenatal diagnosis (Cowan 2008, pp. 93–94; Löwy 2014, p. 293). Also, evidence about the influential role of eugenics can be found in the years that followed. In the 1960s and until the 1970s genetic technological advances which became useful for prenatal testing (Suter 2002, p. 235) were accompanied by eugenics motivations. From the literature we learn that during this period the discovery of DNA inspired eugenic sympathies among geneticists (Kerr and Shakespeare 2002, p. 68) and “throughout the 1960s, most of the leading figures in medical genetics […] bluntly described their work as a form of ‘eugenics’” (Paul 1998, p. 137). In the same period we also encounter a controversial discussion on the idea of a “biological revolution” regarding the creation of a “super-race” while famous geneticists were adopting from less extreme up to absurdly extreme eugenics stands (Kerr and Shakespeare 2002, pp. 69–70). Particularly, regarding prenatal diagnosis, it is interesting to consider Glanville William’s statement in 1964:

[I]t is now quite standard practice in a number of hospitals to terminate pregnancy on eugenic grounds where the woman has caught German measles (rubella) during the first trimester […] because there is then grave danger that the child will suffer from deafness, blindness, heart disease or mental deficiency. Some obstetric surgeons operate purely and simply because of the danger of the child being imperfect… (p. 563)

Therefore, unequivocally, the influence of eugenics was present before and in the beginning of the wide use of prenatal screening technology. By looking into the relevant historical evidence until the 1960s, one discovers that the eugenics movement, either explicitly or implicitly, has played a substantial role in the initiation of prenatal diagnosis techniques’ development alongside scientific interest, curiosity and coincidence and in the absence of a women’s request or the idea of women’s choice empowerment as motivation. This is an important observation because it arguably leads to the conclusion that the scientific research that led to the invention of PST techniques did not aim to empower women’s reproductive choice, instead, considering the involvement of eugenics, in many occasions the incentives for such scientific research were, at least to many, morally questionable.

This does not necessarily suggest that PST technology which resulted from this kind of research is inherently morally problematic. Neither does it suggest that the introduction and the subsequent wide expansion of PST techniques was motivate exclusively by eugenics simply because eugenic ideology continued to be influential. However, the fact that there have been continued ethical concerns regarding women’s choices during the wide use of PST suggests that women’s choice empowerment may not have received the required attention in the development of PST strategies. Following this thought, below I examine whether the idea of women’s choice empowerment was the purpose for the wide and routine use of PST techniques or it was used, purposely to an extent, to satisfy different aims.

## The diffusion of prenatal diagnosis techniques since the 1960s: the starring role that women’s choice empowerment did not play

### Swinging 1960s

Historically, the widespread of prenatal testing coincided with the women’s movement (Suter 2002, p. 236). The “sexual revolution” in the 1960s in conjunction with “second-wave feminism” after 1968 and the changes in women’s status resulted in a general legislative shift towards the right to abortion (Löwy 2014, p. 291). Also, a series of unfortunate events in the 1960s, such as the rubella epidemics, the thalidomide scandal, and the German measles epidemic, seem to have influenced attitudes towards abortion. For example, in 1962, we encounter the case of Sherri Finkbine, a TV presenter from Arizona, who after taking thalidomide in the beginning of her pregnancy, was denied abortion by the local Board. Sherri travelled to Sweden to terminate her pregnancy and when she was back in the United States “she defended her choice in the media and became a national celebrity […] her story actually moved abortion from the margins to the centre of the public debate” (Löwy 2014, pp. 291–292). Also, the same period, doctors’ rising concern about women of lower class who did not have access to safe abortions (Greenhouse and Siegel 2010, pp. 63–67) further contributed to the legalisation of abortion (Löwy 2014, p. 292). In a similar way, attitudes towards pregnancy termination changed in many countries, with the majority of western countries legalising abortion one after the other until the mid-1970s (Löwy 2014, p. 292). Simultaneously, in the mid-1960s researchers achieved prenatal diagnosis of chromosomal anomalies. From the late 1960s physicians were able to detect specific inherited fetal defects, while by the early 1970s, more chromosomal anomalies could be detected (Jacobson and Barter 1967; Nadler 1968 in Löwy 2014, p. 292).

Therefore, according to the sequence of events during the 1960s and 1970s, I would argue that the women’s feminist movement, the incidence of epidemics in conjunction with a change in doctors’ attitudes towards women and reproduction have moulded a general societal and medical shift of approach to women and reproduction. This shift, which triggered legal changes and resulted in the legalisation of abortion in many States, practically allowed and promoted the extensive use of prenatal testing**.** In addition, there are arguments such as the one by Löwy suggesting that the wide expansion of prenatal screening use began as a “result of a partly contingent coming together of three medical innovations-amniocentesis, the study of human chromosomes and obstetrical ultrasound-with a social innovation, the decriminalization of abortion” (2014, p. 290). Similarly, Suter has argued that technological advances including those in prenatal testing and legal changes regarding women’s right to abortion “offered unprecedented reproductive choices for women” (2002, p. 236). She continues saying that “[i]n light of changing social attitudes toward women and reproduction, prenatal testing boomed in the late 1970s and early 1980s” (Nowak 1994, p. 464 in Suter 2002, p. 236). We see then that prenatal testing advances and the decriminalisation of abortion significantly contributed to the increase in women’s reproductive choices, as well as, to the expansion of PST use.

Nevertheless, it is important to note that although the coming together of socio-legal changes, medical advancements and coincidental events at the time significantly contributed both to the empowerment of women’s choices and the expansion of PST use, this does not necessarily imply that the expanded use of PST had the same beneficial effect on women’s reproductive choice. Moreover, this does not imply that such an expansion was what women had asked for. Hence, in what follows, I clarify the contribution of women demands to the expansion of PST, and I identify which other purposes, apart from women’s empowerment, such a wide expansion served.

### The expansion of PST use as a response to an authentic demand for access to prenatal diagnosis: a clarification

Primarily, according to historical evidence, I argue that claims that the massive spread of PST occurred as a response to women’s demand and in favour of reproductive choice empowerment are not entirely convincing because in the emergence of bioethics in the 1960s, feminists’ voices were much underestimated if not ignored (Purdy 1996, p. 4) and reproductive ethics did not really emerge until some 20 years later (Purdy 1996, p. vii). Thus, the validity of such claims is at least questionable since the very representative women’s voices, and especially the one of the feminist movement, were hardly listened to. As Vassy has set it out, “the women who campaigned for the right to have an abortion may not have expected that the outcome would be the legalisation of abortion on medical grounds and, finally the diffusion of prenatal testing techniques” (2006, p. 2043). Essentially, women were campaigning for access to abortion, not for the wide diffusion of prenatal diagnosis. This can be justified if one considers that claims on the part of clinicians and scientists that they were acting in response to women’s demand, only concern women with a family history of hereditary disease or those who already had an affected child (Löwy 2014, p. 293; Seavilleklein 2009, p. 73). In the 1970s, when the clinical use of amniocentesis for fetal aneuploidies started, “only a few pregnant women were aware of the fact that their age put them at greater risk of such anomalies” (Löwy 2014, p. 293). Accordingly, one understands that relating the expanded use of PST with a response to women’s demand is rather an over-generalisation that has very little to do with the reality. What can be supported with relative accuracy is that the legalisation of abortion, which partially resulted from a women’s demand, practically facilitated and promoted the use of prenatal testing.^2^ Thus, in the absence of strong evidence that women explicitly and widely asked for PST, the question that raises is whether, despite women not asking for it, the expansion served the purpose of empowering women’s choices.

### Discovering more purposes behind the veneer of women’s choice empowerment

Pointing to the purposes that the wide expansion of PST served, one observes that women’s empowerment regarding choice, if not entirely absent, certainly, never got the attention it deserved. Notwithstanding information about the reluctance on the part of public authorities to regulate and implement prenatal screening programmes in the fear of eugenics (Harbers 2005, pp. 239–246; Vassy 2006, p. 2046), one also encounters information about governmental organisations, interested sectors of the medical profession and the medical supply industry having initiated such programmes, not in response to women’s demands and reproductive choice, but for their own purposes (Farrant 1985, p. 99; Seavilleklein 2009, p. 72). According to historical and research evidence it has been argued that women’s demands for empowerment were rather used in order to endorse certain prenatal screening strategies (Vassy 2006, pp. 2041–2051; Suter 2002, p. 236).

An example of such evidence constitutes the development of maternal AFP screening for neural defects and amniocentesis for prenatal diagnosis of spina bifida and Down’s syndrome in the UK. According to Farrant’s analysis in the 1970s governmental interest was focused on the expansion of prenatal screening for women over 40 to counterbalance the cost of care for people with Down’s syndrome and spina bifida (Farrant 1985 in Vassy 2006, p. 2043). Particularly, in 1992, Wald provided evidence of the estimate costs saved in one of his reports (Wald et al. 1992). Ιt should be noted that Wald was not just any author. Since the 1970s, Wald’s scientific and Health technological appraisal reports about prenatal diagnosis constituted the basis upon which the initiation and regulation of prenatal screening programmes in the UK were established (Wald et al. 1977, 1998).^3^ Also, Farrant notes that “[o]bstetricians and geneticists wanted to increase their professional prestige, varieties of intervention, and control in pregnancies, and the medical supply business wanted increased profits” (Farrant 1985, pp. 99–103 in Woliver 2002, p. 32). For instance, the market for ultrasound equipment was found to be “among the fastest growing medical instrumentation markets of all time” (Farrant 1985, p. 102 in Woliver 2002, p. 32). The same attitudes were observed in the US too (Kerr and Shakespeare 2002, pp. 70). Initiatives for the expansion of prenatal screening found support from obstetricians, the medical supply industry and pharmaceutical companies (Vassy 2006, pp. 2043).

Additional evidence that professionals have been a driving force for prenatal screening diffusion can be found in a comparative study at the end of the 1980s. In this study of 12 European countries, Reid and Stocking provide evidence that “professionals rather than consumers determined the diffusion of various prenatal tests” (Reid and Stocking 1991 in Vassy 2006, pp. 2043–2044). An interesting example of such evidence constitutes one from France. Vassy has described in detail how biomedical researchers who imported innovative PST techniques, promoted these techniques “to political and administrative decision makers, health professionals, and the general public and lobbied to get the new tests funded with public money” (2006, p. 2049).

According to the historical evidence, it seems reasonable to think that those advocating that the wide promotion of PST use was an act on behalf of pregnant women, they were hiding different purposes, such as financial profit and scientific ambitions, behind the veneer of women’s choice empowerment. I acknowledge that it would be dogmatic and quite unfair to deny all professionals’ arguments and advocate that their efforts towards the expansion of PST were entirely unrelated to an aim for women’s choice empowerment or that claims that such efforts constituted a response to women’s demand are utterly unjustified. However, the moral derailment observed during the wide use of these technologies shows that practically, such efforts had very little to do with women’s choice.

For example, how can we justify that prenatal screening was routinely offered in an attempt to empower women’s choice when there is evidence that clinicians were offering the tests in ways that ‘encouraged’ women to take them, without providing adequate ‘information about the test, the traits that the test could identify and the importance of the anticipating results’ (Marteau 1992; Press and Browner 1994, p. 213)? Additional evidence that the rapid spread and wide use of PST was linked with fears of litigation on the part of clinicians (Powledge 1979, p. 16; Annas 1996, p. S10) further weakens the validity of claims that clinicians were offering prenatal screening to empower women’s choice. Also, such claims seem unjustified if we consider evidence that providers tended to direct women to choose ambiguous prenatal diagnostic techniques in order to collect as much information as possible from each incident. In such cases, the collection of information did not only aim to refine fetal prognosis, but also to further advance knowledge by turning the clinical practice into an experiment (Hogan 2016, pp. 25–27 & 29; Bianchi et al. 1993, p. 549). Ultimately, how can we justify that a women’s demand existed and prenatal screening was widely and routinely offered in an attempt to empower women’s choice when there is empirical research which undermines this claim? Such research provides evidence that many women did not make deliberate decisions and found it difficult to articulate why they had accepted testing (Press and Browner 1997, pp. 980 & 984), that they often did not understand why they were tested (Smith et al. 1994). Essentially, there is evidence that the process of screening did not enable women to gain the understanding vital to make an informed choice (Green et al. 2004; Bernhardt et al. 1998; van den Berg et al. 2005). Accordingly, even if one accepts that the idea of women’s reproductive choice empowerment was not entirely absent from the pool of aims to be achieved through the wide and subsequent routine use of PST technology, what becomes evident from the above is that this idea has never played the starring role.

## Why the current routine offering of PST does not provide adequate protection of women’s choice

Let us now see what the contribution of this historical review can be in solving current and enduring ethical problems and allaying concerns related to women’s reproductive choice limitations when it comes to routine PST plans. Ultimately, with this work I stress the necessity to ‘look back to move forward’. In other words, “we do not just start off with a set of axioms and apply them to particular cases, we also try to learn from experience” (Glover 1998). Respectively, before the introduction and clinical use of new and future PST technologies in the usual routine manner in the name of the axiom of reproductive choice, it is worthwhile exploring past actions, events and motivations which contributed to the consolidation of such routine. Having done this in this paper, I observed that the problem is much deeper and mostly related to the foundation of the establishment of routine and wide use of PST and not only to particular mistakes occurring within this establishment as a result of human faulty perception and action.

Essentially, I argue that the problem is detected in the general perception, which has been taken for granted, that the wide and routine offer of PST is a ‘shelter’ for women’s choice. By analogy, imagine for a moment that the widespread and routine use of PST techniques can be represented by a building which aims to be a shelter for women’s reproductive choice. However, this shelter is not built on foundations that are compatible with women’s reproductive choice. As a result, the building cracks and weakens whatever remedies are tried. Effectively, this analogy shows that trying to support women’s reproductive choices using practices based on unrelated principles of eugenics and scientific advancement, will cause persisting problems in reconciling these two aims.

Beginning with the invention and tracing the development of PST technologies up to their wide and subsequent routine use which reaches today, this historical review has shown that these technologies’ trajectory and their final consolidation as routine part of prenatal care mostly resulted from a combination of different intentions, motivations, influences, and coincidental events and for purposes other than women’s choice empowerment. In my analogy, this combination constitutes the construction specifications for the foundation of ‘the widespread and routine use of PST’. Nevertheless, whereas the foundation meets its construction specifications, these specifications did not prescribe the operation of the building as a shelter capable to provide adequate protection to women’s choice. Effectively, what is meant by this analogy is that whereas the wide and routine use of PST directly satisfied the purposes for which it was built, the idea of women’s choice was instead fitted within a system that was never created for this purpose. Although women’s choice seems to fit in the supposed shelter, the wears and tears on the building, essentially, the perpetuation of concerns around the quality of women’s choice when it comes to PST, shows that women’s autonomy is not well protected in there.

## Conclusion

In this paper, I suggest that by looking back into history, we can learn from the past to improve the future of PST. I suggest that we need to acknowledge the overt incompatibility between women’s choice empowerment and the different purposes for which the wide and routine PST developed. This acknowledgement leads to a lesson to be learnt; that striving to make women’s choice fit within existing routine screening, which had been established upon ideas incompatible with women’s choice empowerment, will continue to be a challenge for this reason. Therefore, I argue that when it comes to new PST technologies, it is worthwhile reconsidering their clinical introduction in the usual way of a wide and routine offer in the name of the axiom of reproductive choice. Instead, if we aim to move in the direction of empowering women’s choices with the use of PST, Ι stress the necessity to refer to this historical lesson when developing relevant policies so as to effectively evaluate which strategy can satisfy this aim.

## Data Availability

Not applicable.

## Footnotes

1 In this paper, I use the abbreviation PST for prenatal screening and testing.

2 1(1)(d) Section of the Abortion Act (1967) (UK) is an illustration of that. While from what has been discussed above, it becomes evident that women had not necessarily linked their demand for access to abortion with access to prenatal testing, such a link seems likely at legal level. Abortion Act (1967) permits abortions when a serious disability is diagnosed. For instance, looking at Section 1(1)(d) of the Act, for a lawful abortion it is required to establish substantial risk for the child to be born to be seriously ‘handicapped’-as indicated in the Section. Given that the means of election to establish such risk is PST, it is plausible to consider PST as a necessary means to justify abortion under certain circumstances. Following the use of PST, women can choose abortion. Essentially, it seems that the Abortion Act (1967) called for and justified the use of PST to some extent.

3 See for instance the “Report of UK collaborative study on alpha-fetoprotein in relation to neural-tube defects” (Wald et al. 1977) in which Wald et al. described the scientific basis of antenatal screening that “led to the first antenatal serum screening programme for birth defects” (Wald et al. 1998, p. 1).
